# Statistical methodology for recurrent events, with application to major trials in heart failure

**DOI:** 10.1186/1745-6215-14-S1-P143

**Published:** 2013-11-29

**Authors:** Jennifer Rogers, Stuart Pocock

**Affiliations:** 1 London School of Hygiene and Tropical Medicine, London, UK

## 

Composite outcomes are frequently adopted as primary endpoints in clinical trials as they consider fatal and non-fatal consequences of the disease under study and lead to higher event rates. Such analyses of time to first event are suboptimal for a chronic disease such as heart failure, characterised by recurrent hospitalisations, as information on repeats is ignored.

We present a comparison of methods of analysing data on repeat hospitalisations, using data from major trials in heart failure. In addition to describing each method and its estimated treatment effect and statistical significance, we investigated statistical power using bootstrapping techniques.

Recurrent heart failure hospitalisations were analysed using the Andersen-Gill, Poisson and Negative Binomial methods. Analyses of recurrent events can be confounded by the competing risk of death. Death was incorporated into analyses by treating it as an additional event in the recurrent event process and by considering methods that jointly model heart failure hospitalisations and mortality. We used a parametric joint frailty model to analyse the recurrent heart failure hospitalisations and time to cardiovascular death simultaneously.

Our analyses show that methods taking account of repeat hospital admissions demonstrate a larger treatment benefit than the conventional time to first event analysis, even when accounting for death. Inclusion of recurrent events also leads to a considerable gain in statistical power compared to the time to first event approach. It seems plausible that in future heart failure trials, treatment benefit would not be confined to first hospitalisations only and so recurrent events should be routinely incorporated.

